# 4-[5-(3-Pyrid­yl)-2*H*-tetra­zol-2-ylmeth­yl]benzonitrile

**DOI:** 10.1107/S1600536808009550

**Published:** 2008-05-03

**Authors:** Bin Hu

**Affiliations:** a Ordered Matter Science Research Center, College of Chemistry and Chemical Engineering, Southeast University, Nanjing 210096, People’s Republic of China

## Abstract

In the title compound, C_14_H_10_N_6_, the pyridine and tetra­zole rings are nearly coplanar and are twisted from each other by a dihedral angle of only 0.86 (9)°. The benzene ring makes a dihedral angle of 70.55 (6)° with the tetra­zole ring.

## Related literature

For the use of tetra­zole derivatives in coordination chemisty, see: Arp *et al.* (2000 ▶); Hu *et al.* (2007 ▶); Wang *et al.* (2005 ▶); Xiong *et al.* (2002 ▶).
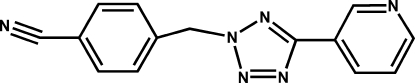


## Experimental

### 

#### Crystal data

C_14_H_10_N_6_
*M* *_r_* = 262.28
Triclinic, 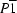

*a* = 8.0452 (16) Å
*b* = 8.7081 (17) Å
*c* = 10.171 (2) Å
α = 94.61 (3)°
β = 104.95 (3)°
γ = 111.11 (3)°
*V* = 630.3 (3) Å^3^
*Z* = 2
Mo *K*α radiation
μ = 0.09 mm^−1^
*T* = 293 (2) K
0.4 × 0.35 × 0.35 mm

#### Data collection

Rigaku Mercury2 diffractometer
Absorption correction: multi-scan (*CrystalClear*; Rigaku, 2005 ▶) *T*_min_ = 0.962, *T*_max_ = 0.968
6638 measured reflections
2882 independent reflections
2063 reflections with *I* > 2σ(*I*)
*R*_int_ = 0.035

#### Refinement

*R*[*F* ^2^ > 2σ(*F* ^2^)] = 0.049
*wR*(*F* ^2^) = 0.123
*S* = 1.04
2882 reflections
181 parameters
H-atom parameters constrained
Δρ_max_ = 0.17 e Å^−3^
Δρ_min_ = −0.20 e Å^−3^

### 

Data collection: *CrystalClear* (Rigaku, 2005 ▶); cell refinement: *CrystalClear*; data reduction: *CrystalClear*; program(s) used to solve structure: *SHELXS97* (Sheldrick, 2008 ▶); program(s) used to refine structure: *SHELXL97* (Sheldrick, 2008 ▶); molecular graphics: *ORTEPIII* (Burnett & Johnson, 1996 ▶) and *ORTEP-3 for Windows* (Farrugia, 1997 ▶); software used to prepare material for publication: *SHELXL97*.

## Supplementary Material

Crystal structure: contains datablocks I, global. DOI: 10.1107/S1600536808009550/dn2333sup1.cif

Structure factors: contains datablocks I. DOI: 10.1107/S1600536808009550/dn2333Isup2.hkl

Additional supplementary materials:  crystallographic information; 3D view; checkCIF report

## Figures and Tables

**Fig. 1.**
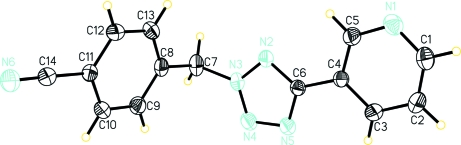
A view of the title compound with the atomic numbering scheme. Displacement ellipsoids were drawn at the 30% probability level. H atoms are represented as small spheres of arbitrary radii.

## supplementary crystallographic information

### Comment

In the past five years, we have focused on the chemistry of tetrazole derivatives because of their multiple coordination modes as ligands to metal ions and for the construction of novel metal-organic frameworks (Wang *et al.*, 2005; Xiong *et al.*, 2002). We report here the crystal structure of the title compound, 4-((5-(pyridin-3-yl)-2*H*-tetrazol-2-yl)methyl)benzonitrile.

There are three rings in the title compound (Fig. 1). The pyridine and tetrazole rings are nearly coplanar and are twisted from each other by a dihedral angle of only 0.86 (0.09) °.The benzene ring makes a dihedral angle of 70.55 (0.06) ° with the tetrazole ring owing to the methylene bridge which forces the two rings to be twisted twisted from each other. In the pyridine ring, the C1=N1 and C5=N1 bond distance of 1.322 and 1.332Å conforms to the value for a C=N double bond, while the C14—N6 bond length of 1.140 Å conforms to the value for a C≡N bond. The bond distances and bond angles of the tetrazole rings are within the usual ranges (Wang *et al.*, 2005; Arp *et al.*, 2000; Hu *et al.*, 2007).

### Experimental

4-((5-(pyridin-3-yl)-2*H*-tetrazol-2-yl)methyl)benzonitrile (3 mmol) was dissolved in ethanol (20 ml) and evaporated in the air affording colorless block crystals of this compound suitable for X-ray analysis were obtained.

### Refinement

All H atoms were fixed geometrically and treated as riding with C–H = 0.93 Å (aromatic) and 0.97 Å (methylene) with *U*_iso_(H) =1.2Ueq(C).

### Crystal data

**Table:**

C_14_H_10_N_6_	*Z* = 2
*M**_r_* = 262.28	*F*_000_ = 272
Triclinic, *P*1	*D*_x_ = 1.382 Mg m^−^^3^
Hall symbol: -P 1	Mo *K*α radiation λ = 0.71073 Å
*a* = 8.0452 (16) Å	Cell parameters from 2882 reflections
*b* = 8.7081 (17) Å	θ = 3.4–27.5º
*c* = 10.171 (2) Å	µ = 0.09 mm^−^^1^
α = 94.61 (3)º	*T* = 293 (2) K
β = 104.95 (3)º	Block, colourless
γ = 111.11 (3)º	0.4 × 0.35 × 0.35 mm
*V* = 630.3 (3) Å^3^	

### Data collection

**Table:**

Rigaku Mercury2 diffractometer	2882 independent reflections
Radiation source: fine-focus sealed tube	2063 reflections with *I* > 2σ(*I*)
Monochromator: graphite	*R*_int_ = 0.035
Detector resolution: 13.6612 pixels mm^-1^	θ_max_ = 27.5º
*T* = 293(2) K	θ_min_ = 3.4º
ω scans	*h* = −10→10
Absorption correction: multi-scan(CrystalClear; Rigaku, 2005)	*k* = −11→11
*T*_min_ = 0.962, *T*_max_ = 0.968	*l* = −13→13
6638 measured reflections	

### Refinement

**Table:**

Refinement on *F*^2^	Secondary atom site location: difference Fourier map
Least-squares matrix: full	Hydrogen site location: inferred from neighbouring sites
*R*[*F*^2^ > 2σ(*F*^2^)] = 0.049	H-atom parameters constrained
*wR*(*F*^2^) = 0.123	*w* = 1/[σ^2^(*F*_o_^2^) + (0.0473*P*)^2^ + 0.0983*P*] where *P* = (*F*_o_^2^ + 2*F*_c_^2^)/3
*S* = 1.04	(Δ/σ)_max_ < 0.001
2882 reflections	Δρ_max_ = 0.17 e Å^−^^3^
181 parameters	Δρ_min_ = −0.20 e Å^−^^3^
Primary atom site location: structure-invariant direct methods	Extinction correction: none

### Special details

**Table:**

Geometry. All esds (except the esd in the dihedral angle between two l.s. planes) are estimated using the full covariance matrix. The cell esds are taken into account individually in the estimation of esds in distances, angles and torsion angles; correlations between esds in cell parameters are only used when they are defined by crystal symmetry. An approximate (isotropic) treatment of cell esds is used for estimating esds involving l.s. planes.
Refinement. Refinement of *F*^2^ against ALL reflections. The weighted *R*-factor *wR* and goodness of fit *S* are based on *F*^2^, conventional *R*-factors *R* are based on *F*, with *F* set to zero for negative *F*^2^. The threshold expression of *F*^2^ > σ(*F*^2^) is used only for calculating *R*-factors(gt) *etc*. and is not relevant to the choice of reflections for refinement. *R*-factors based on *F*^2^ are statistically about twice as large as those based on *F*, and *R*- factors based on ALL data will be even larger.

### Fractional atomic coordinates and isotropic or equivalent isotropic displacement parameters (Å^2^)

**Table:**

	*x*	*y*	*z*	*U*_iso_*/*U*_eq_	
C1	0.1058 (3)	−0.1591 (2)	0.5558 (2)	0.0572 (5)	
H1	0.0253	−0.2512	0.5801	0.069*	
C2	0.2506 (3)	−0.0395 (2)	0.65976 (19)	0.0573 (5)	
H2	0.2661	−0.0497	0.7520	0.069*	
C3	0.3732 (2)	0.0965 (2)	0.62507 (17)	0.0492 (4)	
H3	0.4736	0.1790	0.6936	0.059*	
C4	0.3447 (2)	0.10834 (19)	0.48717 (15)	0.0379 (3)	
C5	0.1926 (2)	−0.0186 (2)	0.39132 (18)	0.0503 (4)	
H5	0.1717	−0.0109	0.2983	0.060*	
C6	0.4708 (2)	0.24879 (19)	0.44434 (15)	0.0389 (4)	
C7	0.6451 (3)	0.4856 (2)	0.2136 (2)	0.0558 (5)	
H7A	0.7176	0.6053	0.2454	0.067*	
H7B	0.5309	0.4698	0.1420	0.067*	
C8	0.7564 (2)	0.4087 (2)	0.15309 (17)	0.0443 (4)	
C9	0.9485 (2)	0.4701 (2)	0.21250 (18)	0.0493 (4)	
H9	1.0070	0.5578	0.2888	0.059*	
C10	1.0544 (2)	0.4029 (2)	0.16004 (17)	0.0474 (4)	
H10	1.1835	0.4445	0.2011	0.057*	
C11	0.9677 (2)	0.2733 (2)	0.04600 (16)	0.0417 (4)	
C12	0.7746 (2)	0.2088 (2)	−0.01356 (17)	0.0513 (4)	
H12	0.7162	0.1201	−0.0892	0.062*	
C13	0.6696 (2)	0.2767 (2)	0.03990 (18)	0.0523 (4)	
H13	0.5403	0.2340	0.0000	0.063*	
C14	1.0826 (2)	0.2107 (2)	−0.01146 (17)	0.0490 (4)	
N1	0.0737 (2)	−0.15148 (18)	0.42275 (17)	0.0583 (4)	
N2	0.45326 (18)	0.26697 (17)	0.31385 (13)	0.0448 (3)	
N3	0.59763 (19)	0.41048 (17)	0.32955 (14)	0.0455 (3)	
N4	0.6996 (2)	0.47768 (19)	0.45939 (16)	0.0551 (4)	
N5	0.61961 (19)	0.37565 (18)	0.53498 (14)	0.0514 (4)	
N6	1.1781 (2)	0.1676 (2)	−0.05615 (16)	0.0654 (5)	

### Atomic displacement parameters (Å^2^)

**Table:**

	*U*^11^	*U*^22^	*U*^33^	*U*^12^	*U*^13^	*U*^23^
C1	0.0591 (11)	0.0495 (10)	0.0713 (13)	0.0216 (9)	0.0306 (10)	0.0206 (9)
C2	0.0666 (12)	0.0641 (12)	0.0495 (10)	0.0281 (10)	0.0255 (9)	0.0203 (9)
C3	0.0496 (10)	0.0522 (10)	0.0430 (9)	0.0188 (8)	0.0125 (8)	0.0065 (7)
C4	0.0371 (8)	0.0419 (8)	0.0386 (8)	0.0188 (7)	0.0144 (7)	0.0053 (6)
C5	0.0526 (10)	0.0491 (10)	0.0444 (9)	0.0150 (8)	0.0159 (8)	0.0054 (7)
C6	0.0354 (8)	0.0438 (9)	0.0394 (8)	0.0169 (7)	0.0140 (7)	0.0029 (7)
C7	0.0618 (11)	0.0595 (11)	0.0698 (12)	0.0333 (10)	0.0402 (10)	0.0299 (9)
C8	0.0468 (9)	0.0490 (9)	0.0487 (9)	0.0224 (8)	0.0255 (8)	0.0201 (8)
C9	0.0491 (10)	0.0486 (10)	0.0475 (9)	0.0146 (8)	0.0186 (8)	0.0044 (8)
C10	0.0364 (8)	0.0534 (10)	0.0496 (9)	0.0144 (8)	0.0144 (8)	0.0074 (8)
C11	0.0406 (9)	0.0491 (9)	0.0384 (8)	0.0182 (7)	0.0154 (7)	0.0109 (7)
C12	0.0438 (9)	0.0598 (11)	0.0427 (9)	0.0165 (8)	0.0093 (8)	0.0009 (8)
C13	0.0371 (9)	0.0664 (11)	0.0541 (10)	0.0197 (8)	0.0157 (8)	0.0137 (9)
C14	0.0450 (9)	0.0571 (10)	0.0426 (9)	0.0189 (8)	0.0125 (8)	0.0064 (8)
N1	0.0565 (9)	0.0442 (8)	0.0663 (10)	0.0107 (7)	0.0201 (8)	0.0074 (7)
N2	0.0430 (8)	0.0487 (8)	0.0437 (8)	0.0157 (6)	0.0188 (6)	0.0089 (6)
N3	0.0428 (8)	0.0474 (8)	0.0531 (8)	0.0184 (7)	0.0246 (7)	0.0121 (7)
N4	0.0447 (8)	0.0538 (9)	0.0591 (9)	0.0095 (7)	0.0194 (7)	0.0059 (7)
N5	0.0437 (8)	0.0523 (8)	0.0502 (8)	0.0097 (7)	0.0162 (7)	0.0055 (7)
N6	0.0581 (10)	0.0839 (12)	0.0611 (10)	0.0351 (9)	0.0227 (8)	0.0040 (9)

### Geometric parameters (Å, °)

**Table:**

C1—N1	1.322 (2)	C7—H7B	0.9700
C1—C2	1.369 (3)	C8—C9	1.382 (2)
C1—H1	0.9300	C8—C13	1.389 (3)
C2—C3	1.381 (3)	C9—C10	1.377 (2)
C2—H2	0.9300	C9—H9	0.9300
C3—C4	1.380 (2)	C10—C11	1.383 (2)
C3—H3	0.9300	C10—H10	0.9300
C4—C5	1.381 (2)	C11—C12	1.389 (2)
C4—C6	1.461 (2)	C11—C14	1.443 (2)
C5—N1	1.332 (2)	C12—C13	1.380 (2)
C5—H5	0.9300	C12—H12	0.9300
C6—N2	1.3265 (19)	C13—H13	0.9300
C6—N5	1.348 (2)	C14—N6	1.140 (2)
C7—N3	1.464 (2)	N2—N3	1.3277 (19)
C7—C8	1.509 (2)	N3—N4	1.315 (2)
C7—H7A	0.9700	N4—N5	1.322 (2)
			
N1—C1—C2	123.89 (17)	C9—C8—C7	119.23 (16)
N1—C1—H1	118.1	C13—C8—C7	121.38 (16)
C2—C1—H1	118.1	C10—C9—C8	120.79 (16)
C1—C2—C3	118.73 (17)	C10—C9—H9	119.6
C1—C2—H2	120.6	C8—C9—H9	119.6
C3—C2—H2	120.6	C9—C10—C11	119.63 (15)
C4—C3—C2	119.00 (17)	C9—C10—H10	120.2
C4—C3—H3	120.5	C11—C10—H10	120.2
C2—C3—H3	120.5	C10—C11—C12	120.21 (15)
C3—C4—C5	117.22 (15)	C10—C11—C14	118.62 (15)
C3—C4—C6	121.38 (15)	C12—C11—C14	121.14 (15)
C5—C4—C6	121.40 (14)	C13—C12—C11	119.73 (16)
N1—C5—C4	124.61 (16)	C13—C12—H12	120.1
N1—C5—H5	117.7	C11—C12—H12	120.1
C4—C5—H5	117.7	C12—C13—C8	120.24 (16)
N2—C6—N5	112.35 (14)	C12—C13—H13	119.9
N2—C6—C4	124.60 (14)	C8—C13—H13	119.9
N5—C6—C4	123.05 (14)	N6—C14—C11	177.31 (19)
N3—C7—C8	111.58 (13)	C1—N1—C5	116.53 (16)
N3—C7—H7A	109.3	C6—N2—N3	101.60 (13)
C8—C7—H7A	109.3	N4—N3—N2	114.04 (13)
N3—C7—H7B	109.3	N4—N3—C7	122.32 (15)
C8—C7—H7B	109.3	N2—N3—C7	123.60 (15)
H7A—C7—H7B	108.0	N3—N4—N5	106.02 (13)
C9—C8—C13	119.38 (16)	N4—N5—C6	105.98 (13)
